# Newly modified ‘pseudo flap’ without compromising vascularity to enhance repair of long distal ureteral loss: A retrospective analysis of a prospective database

**DOI:** 10.1002/bco2.327

**Published:** 2024-02-20

**Authors:** Majid Mirzazadeh, Merhan Badran, Whitney Smith

**Affiliations:** ^1^ Department of Urology, Wake Forest University School of Medicine Wake Forest Baptist Medical Center Winston‐Salem North Carolina USA; ^2^ Department of Urology Thomas Jefferson University Philadelphia Pennsylvania USA

**Keywords:** Boari, pseudo‐flap, psoas hitch, ureteric injury, ureteroureterostomy

## Abstract

**Objective:**

To present an alternative technique called pseudo‐flap for reconstructing long ureteral defects as an alternative to Boari flap. Despite being used for more than 70 years by urologists for tension‐free reconstruction of distal and mid‐ureteral defects, the Boari flap exhibits high complication rates, with an average of 27% (range 5.5%–30.4%). These complications arise from compromised blood supply, attributed to incisions made on all three sides of the flap and dependence on the flap base as the sole source of blood supply.

**Methods:**

We retrospectively reviewed patients who underwent our modified technique by a single surgeon between 2008 and 2021. We used a semi‐oblique cystotomy on the lowest part of the anterior and contralateral aspects of the bladder after complete release from adhesions and sacrificing the superior vesical pedicle, if necessary. The innovative part of the technique involved making short relaxing incisions at different levels on both sides of a pseudo‐flap while pushing the bladder dome upward to reach the healthy ureter in a tension‐free manner, followed by anastomosis with a non‐refluxing or refluxing technique.

**Results:**

Fifteen patients underwent the pseudo‐flap technique with a mean follow‐up of 16.9 months. Four had prior radiation, three had hyperthermic intraperitoneal chemotherapy (HIPEC) for peritoneal carcinomatosis, and one had a ureteral stricture in a transplanted kidney. Eight procedures were performed during intraoperative consultations.

Only one patient (7%) developed a major complication (Clavien–Dindo grade ≥2). This patient developed postoperative leak, and none developed obstructive hydronephrosis, suggesting stricture or flap ischemia. The mean length of the flap was 9.3 cm.

**Conclusion:**

Our pseudo‐flap technique has lower complication rates than the traditional Boari flap. It is not technically challenging, minimally compromises blood supply and is thus especially suitable for complex, highly morbid patients with decreased tissue vascularity, such as those with prior radiation and peritoneal carcinomatosis.

## 1 INTRODUCTION

Ureteral injuries have always been challenging to the surgical team. Based on the multitude of primary aetiologies, the length and the location of the ureteric loss, management of ureteral injuries can be a dilemma. Because longer and more proximal ureteral segments cannot be merely repaired with end‐to‐end anastomosis or ureteral reimplant without considerable tension, many other methods for reconstruction have been explained. Techniques for repairing ureteral injuries are numerous and versatile, including direct ureteral reimplant into the bladder dome, ureteroureterostomy, psoas hitch, Boari flap, autologous grafts,^1^ ileal interposition,^2^ transureteroureterostomy,^3^ bridging the gap with appendix or ovarian tube and on‐lay flap with buccal or lingual mucosa; all have been described with different success rates.

Bladder flap was first described by the Italian Urologist Achille Boari in 1894 in canine models,^4^ and even before that by Van Hook in cadavers.^5^ This technique was implemented in patients for the first time in 1963 by Ockerblad.^6^, ^7^ The Boari flap, or ureteroneocystostomy using a raised bladder flap, is still widely used by urologists for the reconstruction of ureteric injuries in both longer segments^8^ and further locations up to mid and proximal ureter,^9^ with relatively favourable outcomes in long‐term follow‐up. Despite the versatility of its indications and common use in urological surgeries, there is currently scarce literature considering the outcomes and complications of the Boari flap procedure.^10^, ^11^ Complications, mostly anastomotic stricture and leakage, can arise from the technique itself in which incision is made on three sides of the flap and blood supply is exclusively driven from the flap's base. Other reasons for complications are various patient risk factors, such as previous radiation exposure, active cancer and unsuccessful prior procedures. These factors interfere with the proper blood supply and healing process, ultimately leading to a higher rate of failure.

Management of complications after the Boari flap is usually very challenging and includes re‐doing Boari flaps^11^ ileal ureter, new techniques for ureteral grafts^12^ or permanent stents or nephrostomy tubes and nephrectomy as the last resort.

To prevent complications of the Boari flap, especially stricture formation, we developed a pseudo‐flap technique, which is a modification of previous procedures. We have been applying it to all patients who potentially need the Boari flap in the past several years.

Our technique, invented by the senior author, is a modification of the psoas hitch technique, described by Turner‐Warwick.^13^ In his psoas hitch technique, Turner‐Warwick was making a few centimetres long, oblique incision in the lowest part of the bladder on the contralateral side of the flap and pushing the bladder dome upward from inside to elevate the bladder dome in the ipsilateral side, where it can be hitched to the psoas muscle. Unfortunately, the length acquired by this method, on many occasions, is not long enough. The short releasing incisions in both sides, wherever the flap is under tension, increase the length of the pseudo‐flap significantly without compromising its blood supply. This is contrary to the Boari flap technique in which incisions in three different sides of the flap leave the flap solely dependent on its base for blood supply. Here, we aim to describe the pseudo‐flap technique and retrospectively review the outcomes in our very complicated population of patients who underwent our modified technique and compare these outcomes with reported series in the literature.

## 2 MATERIALS AND METHODS

We retrospectively reviewed medical charts of all patients who underwent our modified technique in the Department of Urology at Wake Forest School of Medicine performed by a single surgeon from 2008 to 2018. Our cohort were consecutive patients, with our inclusion criteria being whenever ureteral reimplantation with traditional Boari flap or psoas hitch has not been possible and another method of ureteral reconstruction had to be resorted to. As a salvage technique, the exclusion criteria for this procedure are limited to cases where easier alternatives such as reimplantation with a traditional Boari flap or psoas hitch were feasible.

Patient demographics and characteristics are summarized in Table 1. The cohort is generally typical of highly complex patients, which we define as a patient population in which ureteric reimplantation using conventional methods to replace ureteral loss would generally have a higher risk of failure. This complex patient condition can result from various conditions that affect tissue vascularity, and thus later viability of flaps used in reconstruction, as previous history of malignancy, radiation, chemotherapy and so forth.

**TABLE 1:** Patients' demographics, characteristics, and surgical indications.

Characteristic	Value
Personal characteristics	
Total number of patients, *n*	15
Females, *n* (%)	11 (73.33)
Age in years, mean (range)	58.5 (29–89)
BMI in kg/m^2^, mean (range)	27.9 (16.7–50.9)
Comorbid conditions	
Prior transplant procedures, *n* (%)	1 (7)
HIPEC patients with peritoneal carcinomatosis, *n* (%)	3 (20)
Prior radiation history, *n* (%)	4 (27)
Follow‐up in months, mean (range)	16.9 (1–60)
Patients lost to follow‐up, *n* (%)	1 (6.67)
Left side procedures, *n* (%)	8 (53)
Intra‐operative consultations, *n* (%)	8 (53.33)
Ureteral injuries during hysterectomy, *n*	2
Ureteral resection for non‐genitourinary malignancy, *n*	6
Elective/planned procedures, *n* (%)	7 (46.67)
Primary procedure for urothelial cancer of the ureter, *n*	1
Native ureteral stricture, *n*	5
Transplant ureteral stricture, *n*	1

Abbreviation: HIPEC, hyperthermic intraperitoneal chemotherapy.

Follow‐up visits after surgery were performed at 3 months, 12 months and then annually. The patient's symptoms, voiding history, post‐void residual urine and renal ultrasound were performed during each visit. Many patients with cancer history had multiple imaging in between for surveillance of their cancers. Failure of the flap was defined as postoperative leak or obstructive hydronephrosis, which would suggest flap ischemia or stricture reformation.

### 2.1 Surgical technique

In the supine position, a low midline incision is made to expose the normal ureter, which is then dissected down to the fibrotic ureter. To aid in the orientation of the distal ureter during anastomosis and prevent rotation, a marking suture is passed at the 12 o'clock position. Next, the most distal part of the healthy ureter is clipped and cut. The bladder is filled with 300 mL of saline, and the distance between the normal ureter and the bladder is measured. This measurement determines the extent of dissection on the contralateral side of the bladder.

The anterior surface of the bladder down to the bladder neck is dissected and freed. The ligation of the superior vesical pedicle on the contralateral side is optional and mainly dependent on the length of the gap.

**FIGURE 1:**
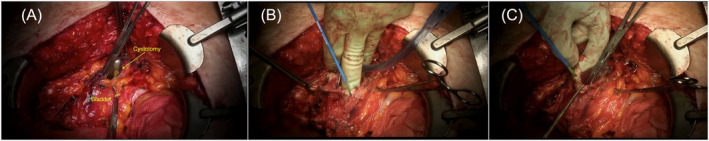
(A) The cystostomy is performed by making an approximately 5‐cm curvilinear incision in the anterior surface of the contralateral side of the bladder after dissecting and freeing the anterior surface of the bladder down to the bladder neck. (B & C) While attempting to reach the bladder flap to the most distal part of the healthy ureter, we detect a tethering point and make a 5‐mm incision on one side at this exact point and another 5‐mm incision on the opposite side. Another set of incisions is made 1–2 cm above the previous ones to eventually reach the bladder to the normal ureter without tension.

**FIGURE 2:**
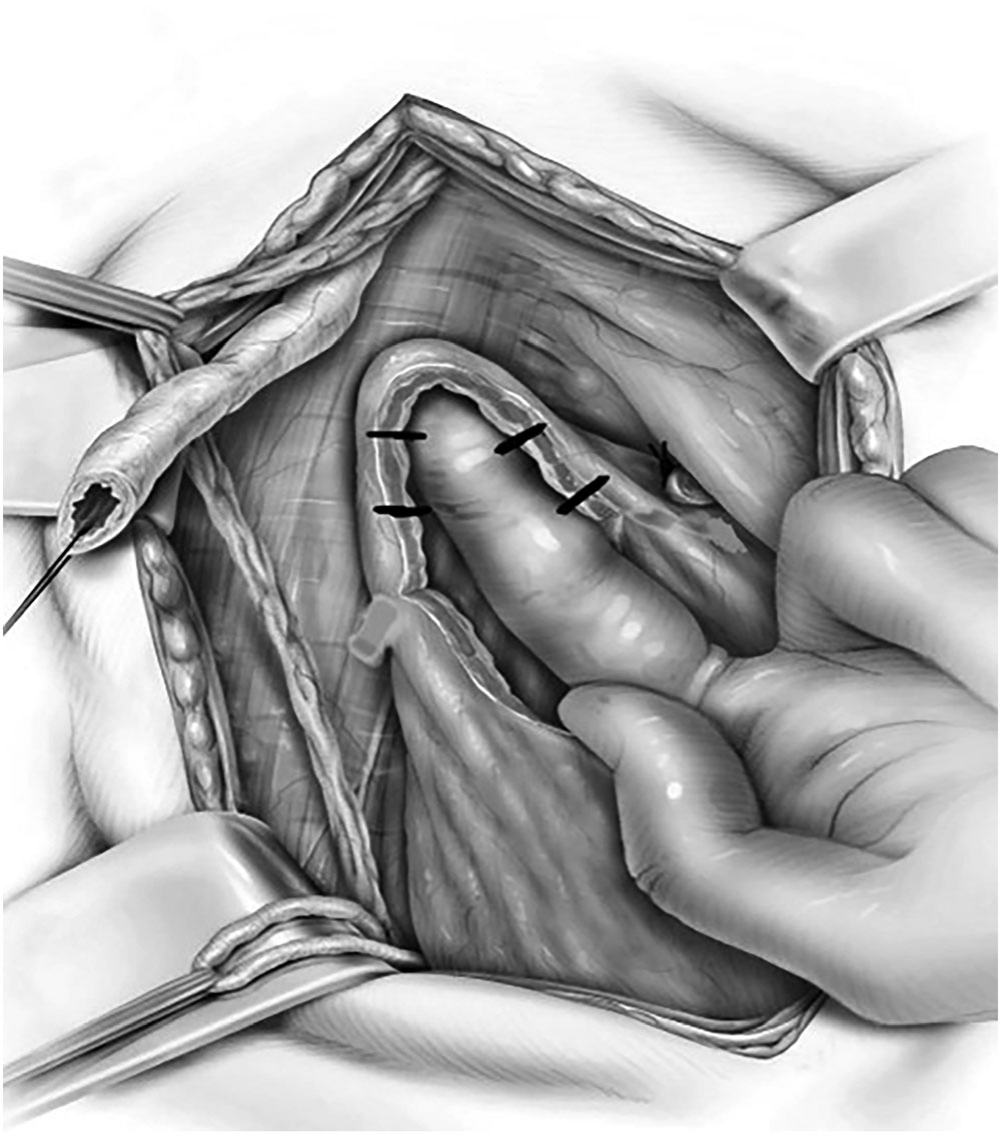
The surgeon's finger is inserted in the bladder dome area in an attempt to reach the uppermost part of the bladder to the healthy ureter. If the gap between the bladder and the most distal part of the healthy ureter is long, we make sets of oppositely situated 5‐mm incisions on both sides of our bladder flap to bridge the gap without tension.

A curvilinear incision of 5 cm is made in the lowest part of the anterior surface on the contralateral side (Figure 1A). Using a finger in the bladder dome area, we attempt to reach the uppermost part of the bladder to the healthy ureter (Figure 2). If the gap is long, we detect tethering points in the bladder wall, which prevent complete bridging of the gap, by pushing the bladder toward the ureter. We make a 5‐mm releasing incision on one side in a tethering point, and then, if needed, another 5‐mm incision on the opposite side a few millimetres higher. If more length is needed, then another set of incisions is made 1–2 cm above the previous ones. This process of making small releasing incisions continues until the bladder can reach the normal ureter without tension (Figure 1B,C).

**FIGURE 3:**
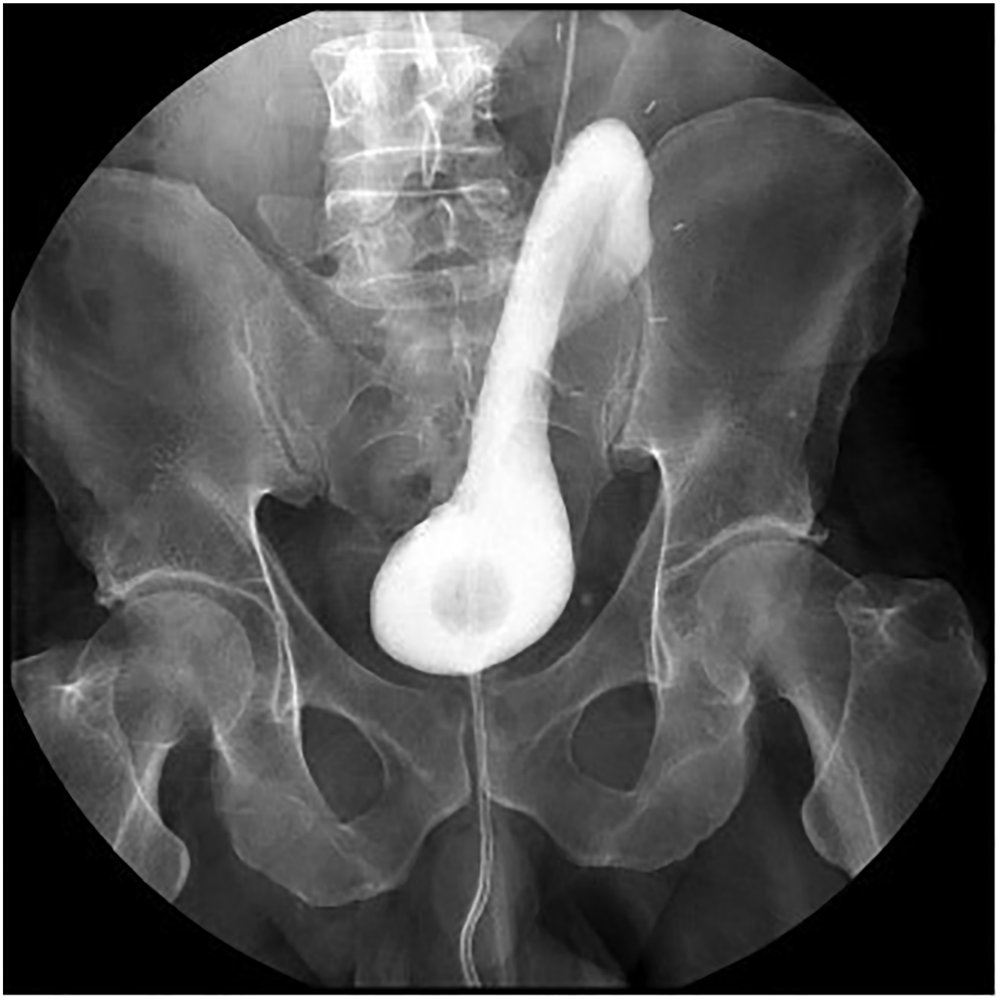
This is a cystogram done 2 weeks after using the bladder pseudo‐flap to replace a lost ureteric segment. The cystogram shows an intact pseudo‐flap with no leakage and thus a successful repair. At this point, the Foley catheter can be removed.

The anastomosis can be either refluxing or non‐refluxing. We typically use the refluxing technique, which involves spatulation of the distal ureter at the 12 o'clock position and passing it through a buttonhole created just distal to the uppermost part of the pseudo‐flap. After performing the stented anastomosis, the pseudo‐flap is secured to the psoas minor tendon using two 2/0 PDS sutures. At this point, the pseudo‐flap is tabularized. Once tabularization is complete, the pseudo‐flap appears as a fully tabularized flap, and the rest of the bladder is repaired in two layers using 2/0 Vicryl stitches. A Foley catheter remains in place for 2–3 weeks and is removed after a cystogram (Figure 3) shows no signs of leakage. A drain is left in, and the patient is discharged the following day.

## 3 RESULTS

We utilized the pseudo‐flap technique in a total of 15 patients between 2008 and 2018. Among the patients, four were male and 11 were female. Their ages ranged from 29 to 89 years, with a mean age of 58.5 years. The mean body mass index (BMI) was 27.9 kg/m^2^, ranging from 16.7 to 50.9. In eight out of the 15 cases, intraoperative consultations were performed without preoperative planning. Two of these consultations were for hysterectomy patients, and the other six were for surgeries related to non‐genitourinary malignancies treated with hyperthermic intraperitoneal chemotherapy (HIPEC). The remaining seven patients underwent elective procedures, consisting of five surgeries for native ureteral strictures, one for a stricture in a transplanted ureter and one for a ureteral defect resulting from urothelial cancer in the distal ureter.

Among the patients, eight (53%) had comorbid conditions: Four patients (27%) had prior radiation, three (20%) underwent concomitant HIPEC for peritoneal carcinomatosis during the surgery and one (7%) required repair of a transplant ureteral stricture. The remaining seven patients did not have any preoperative confounding issues. In our cohort, the mean length of the ureteral defect was 9.3 cm, ranging from 3 to 16 cm. This is also the final length of the pseudo‐flap obtained by the multiple small incisions done in our technique. We measured the gap from the distal part of the healthy ureter to the dome of the bladder; the real ureteral defect was significantly longer. There is no specified width of the flap because we did not make an incision around it. Furthermore, the diameter of the pseudo‐flap is dependent on the number of small incisions made, with more incisions resulting in a longer flap and smaller diameter. The mean follow‐up period for our patients was 16.9 months, ranging from 1 to 60 months. Except for one patient, all patients had at least 6 months of follow‐up. This patient had a follow‐up only at 1 month but could not make it to the second follow‐up and died because of complications from widespread cancer. Our follow‐up protocol included a voiding trial and measurement of residual urine at the time of Foley catheter removal, which occurred 2–3 weeks after surgery. Stent removal visits were scheduled for 4–6 weeks after surgery. Subsequent follow‐up visits were performed at 3 months, 12 months and then annually. During each visit, we assessed the patient's symptoms, voiding history and post‐void residual urine and conducted a renal ultrasound. Patients with a history of cancer often had multiple imaging sessions in between visits for cancer surveillance. Renal scans were reserved for patients who developed de novo hydronephrosis after surgery, which did not occur in our patients. The Clavien–Dindo grading system^14^ was used for reporting postoperative complications. In the follow‐up period, one patient (7%) developed postoperative leak (Clavien–Dindo grade 3), a complication not uncommon following ureteric reimplantation according to the EAU guidelines panel assessment, and was appropriately managed with nephrostomy^15^ that was removed later and did not develop complications on long‐term follow‐up. None of our patients developed obstructive hydronephrosis (Clavien–Dindo grade 2–3), suggesting anastomotic stricture or flap ischemia. Moreover, none of our patients experienced urinary retention, severe frequency, urgency or urinary incontinence (Clavien–Dindo grade 1) after surgery. These findings are consistent with the literature, indicating that bladder function remains independent of its configuration, and the bladder retains its functionality even after reconfiguration. Considering our highly complex patient population, characterized by long ureteric stricture lengths (mean of 9.3 cm) and a lengthy follow‐up period (mean of 16.9 months), we believe that our modification demonstrates reliability and yields favourable outcomes. Our approach exhibits lower complication rates compared with the traditional Boari flap technique and is suitable for complex patients with compromised tissue vascularity resulting from factors such as previous surgeries, concomitant chemotherapy, radiation or renal transplantation.

## 4 DISCUSSION

Repairing long ureteral strictures has always presented a challenge, leading to the development of numerous techniques for this purpose. These methods range from straightforward approaches like direct ureteroureterostomy and ureteroneocystostomy to more complex techniques such as psoas hitch, Boari flap, transureteroureteral anastomosis, autologous grafts from oral mucosae (buccal and lingual mucosae), urogenital mucosae (renal pelvis wall, penile/preputial skin and vein), ureteral replacement with appendix or ileum and potential future options like ureteral tissue engineering,^1^ as well as renal autotransplantation.

The Boari flap has been widely used as a method of ureteroneocystostomy for bridging longer segments of the ureter.^8^, ^9^ However, there is limited literature available on surgical outcomes associated with this technique.^10^, ^11^ Reported complications include urinary leak, uroperitoneum (Clavien–Dindo grade ≥2) and uretero‐vaginal and uretero‐enteric fistula (Clavien–Dindo grade ≥3), as well as late complications such as anastomotic strictures with recurrent hydronephrosis (Clavien–Dindo grade ≥3), which may require permanent stents, nephrostomy tubes or even nephrectomy.^10^, ^11^, ^16^

Various authors have documented their experiences with the Boari flap technique, providing insights into outcomes, success rates and complication rates. In Table 2, we have compiled a comprehensive list of the most relevant papers in this regard. In a study conducted by Kunj Jain et al. in 2022, the authors examined 50 patients who underwent ureteral reimplantation with the Boari flap and reported an 88% success rate, with six documented cases of failure. Surgical ‘failure’ was defined as the presence of flank pain or imaging evidence of ureteral obstruction, the need for or presence of hardware despite undertaking the procedure or the requirement for repeated ureteral reconstruction.^10^

**TABLE 2:** Studies reporting ureteral injury and repair techniques.

Authors	Matthew Lee et al.^17^	Brian W Chao et al.^18^	Xinfei Li et al.^19^	Alsaadi et al.^20^	Farzad Allameh et al.^21^	Abdel Bakayoko et al.^22^	Riedmiller et al.^23^	Gozen et al.^24^	Helfand et al.^25^	Wenske et al.^26^	Ghosh et al.^27^	Ahn et al.^28^	Alex Federico et al.^16^	Ben V. Sionov et al.^11^	Kunj Jain et al.^10^	Benson et al.^9^	Yucai Wu et al.^12^
Total patients, *n*	105	26	42	1	1	1	181	24	13	100	22	24^q^	46	2	50	18	6
Total undergoing Boari flap, *n*	8	9^d^	6	1	1^i^	1^j^	N/A	9^n^	1	18	2	N/A	N/A	2	50	6	N/A
Approach (Open, LS, RUR)	RUR	RUR		N/A	Open	LS	Open	LS	Open	Open	LS	Open	Open	Open	RUR	N/A	LS
Other techniques used^†^, *n* (%)																	
UU	7		6						1		3						
UC	3																
PP	40		4														
Buccal mucosa UP	21																
Lingual mucosa UP			8														
UP with bladder onlay flap by NTTAA																	6
Appendiceal bypass	1		7														
Ileal ureter			9													10	
Side‐to‐side reimplant	7																
End‐to‐end reimplant	26												(37)				
Ureteral tapering then reimplant											2						
Auto‐transplantation																2	
Psoas hitch	10						‐‐^l^	10		58	15		(63)				
Downward nephropexy	7																
UNC		17^e^							6	24							
Balloon dilation			2														
Lich‐Gregoir EVUR								5									
Pyelovesicostomy									5								
Length of ureteral injury in cm																	
*n*		‐‐		‐‐^h^	20	‐‐	‐‐				8, 9			2, N/A^u^		‐‐^x^	
Median	2		3.5														
IQR	1–3																
Range			1–30					1–6	1–7								
Mean								2.92	3.3			5.4			5.54		2
L																	
H												14.5					
Ureteral defect due to^†^, *n* (%)																	
RUS after endoscopic UR, *n* (%)	(49.5)							Yes^m^									
RUS after open UR, *n* (%)	(25.7)						Yes^l^, ^m^	Yes^m^	13^o^					2^v^			6^y^
RUS after both endoscopic and open UR, (%)	(24.8)																
Iatrogenic injury^a^, *n*		15					Yes^m^	Yes^m^		Yes^m^	Yes^m^	11	Yes^m^		(16)	Yes^m^	
Stricture after radiotherapy							Yes^m^						12^s^				
Stricture after IPC, *n*						1											
Stricture in transplant ureter, *n*				1													
Stricture after lithotripsy, *n*			42^f^		1							12^r^					
Stricture after inflammation^b^							Yes^m^	Yes^m^		Yes^m^	Yes^m^					Yes^m^	
Stricture after tumours								Yes^m^		Yes^m^, ^p^			Yes^m^		(22)	Yes^m^	
Stricture after stone								Yes^m^			Yes^m^					Yes^m^	
Stricture after fistulas^c^										Yes^m^							
Stricture after tuberculosis											Yes^m^						
Congenital stricture																	
Trauma to ureter												1					
Follow‐up period in months																	
*n*				5	8	3							76	120, 24			
Median	15.1	21	27.3					35	41	48.7							
IQR	5.0–30.4	12–30															
Range								3–62		12.3–253		1–122					
Mean							54					32.75			16.28	57.6	24.5
L			10.1														
H			44.5				114										
Success rate, %	95	92.3	97.6	‐‐		‐‐	96.7	95.8	‐‐	‐‐		‐‐	‐‐	‐‐	88	94	83.3, 100^z^
Complications^†^, *n*																	N/A
Minor (Clavie*n* ≤ 2)																	
Prolonged ileus								1									
DVT								1									
Recurrent lower UTIs, Urosepsis							5			Yes^m^	3	1				Yes^m^	
Major (Clavie*n* > 2)*, *n* (%)	2 (1.9)														3 (6)^w^		
IO bowel injury								1				1					
IO iliac vein injury												1					
FAILURE															6 (12)		
RUS, *n* (%)		2 (7.6)		1 (100)			4 (2)	2 (8.3)	2 (15.3)								
VUR		10					2				1						
Urinary leak, *n* (%)													9 (19.5)^t^				
HN, *n* (%)			1 (100)^g^		1 (100)					20 (20)			3 (6.5)			1 (5.5)	
Unilateral renal impairment													2				

Abbreviations: EVUR, extravesical ureteral reimplantation; HN, hydronephrosis; IO, Intraoperative; IPC, Intraperitoneal chemotherapy; LS, laparoscopic; N/A, not available; PP, pyeloplasty; RUR, robotic ureteral reconstruction; RUS, recurrent ureteral stricture; UC, ureterocalicostomy; UNC, ureteroneocystostomy; UP, ureteroplasty; UR, ureteral reconstruction; URS, ureteroscope; UTI, Urinary tract infection; UU, ureteroureterostomy; VUR, vesicoureteral reflux.

^a^ Gynaecologic surgery was reported as the most common iatrogenic cause of ureteral injury. Other iatrogenic causes include previous endoscopic procedures involving the ureter and general surgery for colonic tumours or pelvic sarcomas.

^b^ According to reports, inflammatory causes that led to ureteral strictures are appendicitis, retroperitoneal fibrosis, endometriosis and amyloidosis.

^c^ Fistulas reported are ureterovaginal mainly. Others include ureterocolonic, ureterocutaneous, ureterouterine and vesicocolonic.

^d^ Adjunctive psoas hitch or Boari flap was performed in these nine patients in addition to robotic ureteroneocystostomy with side‐to‐side anastomosis. The separate number of those who underwent the Boari flap is not mentioned.

^e^ Here ureteroneocystostomy was done with side‐to‐side anastomosis.

^f^ Patients in this cohort underwent ureteroscopic holmium laser lithotripsy.

^g^ This one patient developed hydronephrosis after ureteroureterostomy.

^h^ The authors mentioned that the ureteral defect was ‘distal’ extending to the vesicoureteral junction (VUJ) but did not state the precise length in cm.

^i^ Here, the patient underwent a Boari flap in addition to psoas hitch.

^j^ The authors here described a modified Boari flap.

^k^ The authors mentioned that another technique used was psoas hitch ureteroneocystostomy but did not specify the exact number of patients on which this other technique was implemented.

^l^ Here, indications for previous surgeries causing ureter loss were the correction of congenital anomalies, reflux therapy and ureteroneocystostomy.

^m^ The authors did not mention numbers for patients suffering from each respective cause or complication.

^n^ Here, all patients who underwent Boari flap had vesicopsoas hitch done in addition.

^o^ Here, recurrent ureteral strictures occurred after open reconstruction for transplant ureteral strictures.

^p^ Here, the most common malignancy leading to ureteral stricture was transitional cell carcinoma (TCC).

^q^ The authors here described a surgical modification performed on all their 24 patients which entailed avoiding extending bladder cystotomy to the dome, so the surgeon would insert fingers into the bladder and later fixation to the ipsilateral psoas.

^r^ Of those 12 patients, eight had planned ureteral resection: four due to transitional cell carcinoma and the other four due to other tumours (sarcoma and sigmoid adenocarcinoma) and endometriosis affecting the ureter. Of the remaining four, two developed strictures due to nephrolithiasis, and one had a ureteral stricture in addition to ‘chronic flank pain and aplastic anaemia’.

^s^ Eight of these 12 patients who underwent radiotherapy also underwent neoadjuvant chemoradiotherapy.

^t^ A urinary leak occurred in nine patients: five uretero‐vaginal fistula, three uroperitoneum and one uretero‐enteral fistula.

^u^ The length of the ureteral defect was 2 cm in one patient and not mentioned in the other.

^v^ The recurrence of ureteral strictures here was after doing previous psoas hitch and Boari flap for sigmoid and rectal adenocarcinomas involving the ureter.

^w^ Three patients experienced major complications (Clavien–Dindo grade ≥2) in the 30‐day postoperative period, with one grade IIIa (urine leak required drainage via interventional radiology [IR]) and two grade IIIb (IR embolization of pseudoaneurysm, colovesical fistula repaired with clip).

^x^ The authors did not mention precise length but stated varying lengths of ureter often “just distal to the renal pelvis” for lengths of ureteric defects they reported.

^y^ One of these patients had recurrent ureteral stricture after being operated upon by Boari flap.

^z^ Objective success rate (CT urography with improved hydronephrosis) occurred in 83.3% of patients. Subjective success rate (relief of flank pain) occurred in 100% of the patients.

* Following the Boari flap, based on our literature review, the occurrence rate of major postoperative complications (Clavien–Dindo grade ≥2), namely anastomotic leak or ureteral stricture yielding obstructive hydronephrosis with or without subsequent renal impairment, is high, with a calculated average of 26.98% (approximately 27%) and a range from 5.5% to 30.4%. However, it is important to note that the complication rates after utilizing the Boari flap specifically for ureteral reconstruction are difficult to determine because most previous studies state complication rates collectively for all methods employed for ureteral reconstruction and do not report an exact complication rate for each method used.

^†^ Techniques other than the Boari flap, causes of ureter defect and complications were stated here as reported by authors in each respective paper.

In a report by Benson et al. in 1990, the authors shared their experience with ureteric reimplantation in 18 cases, six of which have undergone psoas hitches and Boari flaps. They reported an overall success rate of 94%. In the psoas hitch/Boari flap group, one out of six patients (17%) experienced failure of the ureteral reconstruction. The patient developed subsequent hydronephrosis and cortical atrophy (Clavien–Dindo grade 2 or 3a), which were managed with ureteral stents.^9^ This report is advantageous as it attributes complications specifically to the method of ureteral reconstruction used, providing valuable insights into the potential drawbacks of each approach.

Because of the limitations associated with the traditional Boari flap, the necessity for modifications or alternatives has emerged. Several reports have highlighted new or modified techniques. In 2001, Ahn et al. introduced a modified psoas hitch ureteral reimplantation technique that avoids extending the bladder cystostomy to the dome. The authors argued that this modification enables direct finger insertion into the bladder, facilitating its fixation to the ipsilateral psoas tendon. This modification appears to have a positive impact on flap vascularity, viability and the overall surgical field. In their cohort of 24 patients, the authors reported no instances of chronic flank pain, recurrent pyelonephritis, persistent severe hydronephrosis, compromised renal function or the need for reoperation due to complications or repair failure during the follow‐up period.^28^ Our technique represents a further modification of this approach, with the short relaxing incisions at different levels on both sides of the pseudo‐flap and the mobilization of the bladder dome upward providing extra length to reach the healthy ureter in a tension‐free manner.

Other authors have attempted to completely abandon the Boari flap and instead utilized onlay flaps from buccal or lingual mucosa, along with other techniques, to address long segments of the ureter.^1^

Unfortunately, most of the studies in the literature discussing outcomes of the Boari flap or alternative procedures are not without flaws:

1. These studies often report combined outcome results for various approaches, including the Boari flap, to repair ureteral injuries. They generally do not specify the proportion of failures associated with a specific procedure, making it impossible to determine the specific outcomes of the Boari flap (Wenske et al.,^26^ Helfland et al.,^25^ Gozen et al.,^24^ Matthew et al.,^17^ Brian W Chao et al.,^18^ Riedmiller et al.^23^).
2. The follow‐up periods vary significantly among the studies. Some studies, such as Kunj Jain et al. (mean 16.28 months),^10^ Yucai Wu et al. (mean 24.5 months),^12^ Brian W Chao et al. (median 21 months),^18^ Ghosh et al. (mean 25 months)^27^ and Xinfei Li et al. (median 27.3 + 17.2 months),^19^ have relatively long follow‐up periods. However, others, like Alsaadi et al. (1 and 5 months),^20^ Farzad et al. (8 months)^21^ and AbdelBakayko et al. (3 months),^22^ have short follow‐up durations.
3. Most of these studies consist of small case series, such as Ben Sionov et al.,^11^ Yucai Wu et al.,^12^ Alsaadi et al.,^20^ Farzad et al.^21^ and AbdelBakayko et al.^22^
4. The length of the ureteric segment has not been mentioned in many reports, including those by Ben Sionov et al.,^11^ Wenske et al.,^26^ Gozen et al.^24^ and AbdelBakayko et al.^22^

All of these limitations make it exceedingly challenging to make an objective assessment of the success of the Boari flap procedure based on the available literature. It is also imperative to note that accurately establishing the precise complication rates attributable to the specific utilization of the Boari flap is challenging because most studies reported complication rates collectively for all techniques employed for ureteral reconstruction. Nonetheless following the Boari flap, based on our literature review, the occurrence rate of major postoperative complications (Clavien–Dindo grade ≥2), namely anastomotic leak or ureteral stricture yielding obstructive hydronephrosis with or without subsequent renal impairment, is high, with an average of approximately 27% (ranging from 5.5% to 30.4%). This high complication rate may result from incisions made on three sides of the flap, relying solely on the flap base for blood supply and increasing the risk of subsequent flap ischemia. In our approach, we avoid making incisions on all three sides, resulting in improved blood supply. Our series demonstrates a lower incidence rate (7%) of these complications (ureteral stricture and anastomotic leak) compared with the average rate (27%) reported in the reviewed literature.

Based on our findings, we believe that our pseudo‐flap technique serves as a viable alternative to the Boari flap, yielding improved outcomes compared to the series reported in the literature. However, it is important to acknowledge that the generalizability of our results may be limited because of the nature of this case series, which was conducted by a single surgeon on a small number of patients. Additionally, the follow‐up period was relatively short for some patients, primarily because of mortality resulting from underlying comorbidities. We acknowledge that our group of patients is non‐homogenous with different underlying diseases that may interfere with the interpretation of the outcome. However, being applicable in patients having a wide variety of conditions with good outcomes is the hallmark of our proposed surgical technique. Therefore, further research is urgently needed to explore the application of this innovative technique on a larger scale, involving multiple institutions and a larger patient cohort. Furthermore, although all of our patients underwent open surgery, it appears feasible to adapt this surgical technique to laparoscopic or robotic approaches, applying the same principles.

## 5 CONCLUSION

We recommend utilizing the ‘pseudo‐flap’ modification as a replacement for the Boari flap technique. This modification is supported by a solid anatomical foundation, offers favourable long‐term outcomes, can be applied to highly morbid patients with extensive ureteral injuries and is not overly complex from a technical standpoint. However, to establish this procedure as a standard technique for repairing long lower defects in the lower and mid portions of the ureter, further studies involving a larger patient cohort and multiple surgeons are necessary in the future.

## AUTHOR CONTRIBUTIONS

Dr Mirzazadeh developed the modified surgical technique. Dr Whitney Smith gathered data retrospectively from patients' charts who underwent the newly devised technique over 10 years. Dr Badran was responsible for writing the manuscript besides doing a review of reports that found drawbacks to the traditional Boari flap and/or developed alternative methods of ureteral reimplantation.

## CONFLICT OF INTEREST STATEMENT

We, the authors, declare that we have no known competing financial interests or personal relationships that could have appeared to influence the work reported in this paper. We also declare that we have neither received any support for the present manuscript (e.g., funding, provision of study materials, medical writing and article processing charges) in any time limit nor grants or contracts from any entity, royalties or licences, consulting fees, payment or honoraria for lectures, presentations, speakers bureaus, manuscript writing or educational event, payment for expert testimony, support for attending meetings and/or travel, patents planned, issued or pending, participation on a Data Safety Monitoring Board or Advisory Board, Leadership or fiduciary role in other board, society, committee or advocacy group, paid or unpaid. We did not receive stock, stock options, equipment, materials, drugs, medical writing, gifts and other services, and we declare no other financial or non‐financial interests.

## Data Availability

The authors confirm that the data supporting the findings of this study are available within the article and its supplementary materials.

## References

[1] Xiong S , Wang J , Zhu W , Yang K , Ding G , Li X , et al. Onlay repair technique for the management of ureteral strictures: a comprehensive review. Biomed Res Int. 2020;2020:6178286. 32775430 10.1155/2020/6178286 PMC7407031

[2] Pamecha Y , Shelke U , Patil B , Patwardhan S , Kini S . Use of ileum for complex ureteric reconstruction: assessment of long‐term outcome, complications, and impact on renal function. (0974–7796 [Print]). 10.4103/UA.UA_5_18 PMC6194786 30386088

[3] Ehrlich Richard M , Skinner DG . Complications of transureteroureterostomy. J Urol. 1975;113(4):467–473. 1117516 10.1016/s0022-5347(17)59502-6

[4] Boari A . Contribute sperementale alla plastica delle uretere. Atti Accad Med Ferrara. 1894;14:444.

[5] Lloyd G . FR01‐19 The reter king of Chicago: Weller Vanhook's contributions to reconstructive surgery. J Urol. 2019;201(Supplement 4):e250‐e.

[6] Ockerblad Nelse F , Carlson HE . Surgical treatment of uretero‐vaginal fistula1. J Urol. 1939;42(2):263–268.

[7] Graziano ME , Thompson PM . The story of the Boari flap. J Urol. 2008;179(4S):309.

[8] Lee MA‐O , Lee ZA‐O , Koster HA‐O , Jun MA‐O , Asghar AA‐O , Lee RA‐O , et al. Intermediate‐term outcomes after robotic ureteral reconstruction for long‐segment (≥4 cm) strictures in the proximal ureter: a multi‐institutional experience. (2466‐054X [Electronic]). 10.4111/icu.20200298 PMC7801167 33258325

[9] Benson MC , Ring KS , Olsson CA . Ureteral reconstruction and bypass: experience with ileal interposition, the Boari flap‐psoas hitch and renal autotransplantation. J Urol. 1990;143(1):20–23. 2294254 10.1016/s0022-5347(17)39852-x

[10] Jain K , Alter K , Cheng N , Corse T , Krishnan N , Lee M , et al. MP25‐14 A multi‐institutional experience utilizing Boari flap in robotic urinary reconstruction. J Urol. 2022;207(Supplement 5):e433. 10.1089/end.2022.0618 37128188

[11] Sionov BV , Taha T , Preter D , Salbaq R , Engelstein D , Tsivian A . Re‐do Boari flap for recurrent ureteric stricture. Int Braz J Urol. 2021;47:670–673. 33621021 10.1590/S1677-5538.IBJU.2020.0491 PMC7993970

[12] Wu Y , Zhu W , Yang K , Fan S , Guan B , Huang B , et al. Terminal augmented ureteroplasty with bladder onlay flap technique for recurrent distal ureteral stricture after ureteroneocystostomy: an initial case series. Transl Androl Urol. 2021;10(8):3332. 34532257 10.21037/tau-21-252 PMC8421814

[13] Warwick RT , Worth PH . The psoas bladder‐hitch procedure for the replacement of the lower third of the ureter 1. Br J Urol. 1969;41(6):701–709. 5359493 10.1111/j.1464-410x.1969.tb09981.x

[14] Dindo D , Demartines N , Clavien P‐A . Classification of surgical complications: a new proposal with evaluation in a cohort of 6336 patients and results of a survey. Ann Surg. 2004;240(2):205–213. 15273542 10.1097/01.sla.0000133083.54934.ae PMC1360123

[15] Mitropoulos D , Artibani W , Graefen M , Remzi M , Rouprêt M , Truss M . Reporting and grading of complications after urologic surgical procedures: an ad hoc EAU guidelines panel assessment and recommendations. (1873–7560 [Electronic]). 10.1016/j.eururo.2011.10.033 22074761

[16] Federico A , Gallotta V , Foschi N , Costantini B , Conte C , Pinto F , et al. Surgical outcomes of segmental ureteral resection with ureteroneocystostomy after major gynecologic surgery. Eur J Surg Oncol. 2020;46(7):1366–1372. 32278519 10.1016/j.ejso.2020.03.216

[17] Lee M , Lee Z , Okoro C , Asghar A , Lee R , Strauss D , et al. PD35‐02 Multi‐institutional experience with robotic ureteral reconstruction for recurrent strictures after prior failed management. J Urol. 2021;206(Supplement 3):e588‐e. 10.1002/bco2.224 PMC10071084 37025480

[18] Chao BW , Slawin JR , Shakir NA , Kuppa SA , Okoro CU , Harrison R , et al. PD35‐07 Intermediate outcomes following robotic nontransecting ureteral reimplantation. J Urol. 2021;206(Supplement 3):e591‐e.

[19] Li X , Qiao J , Xiong S , Wang J , Wang Q , Li Z , et al. The surgical outcomes of reconstruction for the treatment of ureteral stricture after holmium laser lithotripsy: the comprehensive experiences. Asian J Surg. 2022;45(12):2713–2718. 35346585 10.1016/j.asjsur.2022.03.018

[20] Alsaadi S , Toussi H , Alseiari M , Al Ahmed M , Zaman M . POS‐749 Boari flap ureteric re‐implantation, a salvage procedure for kidney transplant ureteral stenosis. Kidney Int Rep. 2022;7(2):S324.

[21] Allameh F , Hojjati SA , Faraji S , Eslami A , Garousi M . Calicovesicostomy surgery in the patient with ureteral rupture. Urol Case Rep. 2022;44:102133. 35769129 10.1016/j.eucr.2022.102133 PMC9234219

[22] Bakayoko A , El Akri M , Freton L , Hascoet J , Khene Z‐E , Grafeille V , et al. V01‐12 Robot assisted ureteral reimplantation side‐to‐side with Boari flap. J Urol. 2022;207(Supplement 5):e55.

[23] Riedmiller H , Becht E , Hertle L , Jacobi G , Hohenfellner R . Psoas‐hitch ureteroneocystostomy: experience with 181 cases. Eur Urol. 1984;10(3):145–150. 6373299 10.1159/000463777

[24] Gözen AS , Cresswell J , Canda AE , Ganta S , Rassweiler J , Teber D . Laparoscopic ureteral reimplantation: prospective evaluation of medium‐term results and current developments. World J Urol. 2010;28:221–226. 19578856 10.1007/s00345-009-0443-8

[25] Helfand BT , Newman JP , Mongiu AK , Modi P , Meeks JJ , Gonzalez CM . Reconstruction of late‐onset transplant ureteral stricture disease. BJU Int. 2011;107(6):982–987. 20825404 10.1111/j.1464-410X.2010.09559.x

[26] Wenske S , Olsson CA , Benson MC . Outcomes of distal ureteral reconstruction through reimplantation with psoas hitch, Boari flap, or ureteroneocystostomy for benign or malignant ureteral obstruction or injury. Urology. 2013;82(1):231–236. 23642933 10.1016/j.urology.2013.02.046

[27] Ghosh B , Jain P , Pal DK . Managing mid and lower ureteral benign strictures: the laparoscopic way. J Laparoendoscopic Adv Surgical Tech. 2017;28(1):25–32. 10.1089/lap.2017.0256 28825970

[28] Ahn M , Loughlin KR . Psoas hitch ureteral reimplantation in adults—analysis of a modified technique and timing of repair. Urology. 2001;58(2):184–187. 11489694 10.1016/s0090-4295(01)01144-x

